# Impact of COVID-19 pandemic on sleep quality among medical and general science students: King Saud University Experience

**DOI:** 10.12669/pjms.38.3.5171

**Published:** 2022

**Authors:** Sultan Ayoub Meo, Joud Mohammed Alkhalifah, Nouf Faisal Alshammari, Wejdan Saud Alnufaie, Ahad Fahad Algoblan

**Affiliations:** 1 Sultan Ayoub Meo, Department of Physiology, College of Medicine, King Saud University, Riyadh, Saudi Arabia; 2 Joud Mohammed Alkhalifah, Department of Physiology, College of Medicine, King Saud University, Riyadh, Saudi Arabia; 3 Nouf Faisal Alshammari, Department of Physiology, College of Medicine, King Saud University, Riyadh, Saudi Arabia; 4 Wejdan Saud Alnufaie, Department of Physiology, College of Medicine, King Saud University, Riyadh, Saudi Arabia; 5 Ahad Fahad Algoblan, Department of Physiology, College of Medicine, King Saud University, Riyadh, Saudi Arabia

**Keywords:** Sleep health, Science students, Medical students, Pittsburgh Sleep Quality Index, COVID-19

## Abstract

**Objectives:**

Sleep is a vital component for overall health and well-being, and it plays an essential role in social, physical, psychological, and cognitive health. This study aimed to appraise the sleep quality in medical and science students during the COVID-19 pandemic.

**Methods:**

This questionnaire-based cross sectional study was conducted in the Department of Physiology, College of Medicine, King Saud University, Riyadh, Saudi Arabia, during September-December 2020. In this study, a validated self-administered electronic questionnaire was distributed to 1000 students, 782 (78.2%) of whom completed the study. The selection of students was based on using the stratified random sampling. The Pittsburgh Sleep Quality Index (PSQI) instrument scale was used to assess the sleep quality among medical and general sciences students.

**Results:**

Out of 782 respondents, 410 (52.4%) were medical students, and 372 (47.6%) were science students, including Physics, Chemistry, Mathematics, Statistics, Botany, and Zoology. Among the medical students, 143 (34.9%) were in pre-clinical years (1st and 2nd), while 266 (64.9%) of them were in clinical years (3rd, 4th, and 5th). Among all medical and general sciences students, it was found that 669 (85.5%) had poor sleep quality with a mean PSQI global score (mean 8.356) among them 336 (50.2%) were medical, and 333 (49.8%) were science students. Science students’ sleep quality was poorer (mean 8.78) than their medical counterparts (mean= 7.93).

**Conclusion:**

The COVID-19 pandemic has a significant negative impact on students’ mental health and sleep quality. Both medical and general science students showed alarming levels of sleep deprivation and concerning low sleep quality during the COVID-19 pandemic. The sleep deprivation among students may be due to the sudden change of pedagogy in education driven by the COVID-19 pandemic. Sleep quality is quite a critical issue to be evaluated and addressed nationally and globally.

## INTRODUCTION

 The “Severe Acute Respiratory Syndrome Coronavirus-2 (SARS-CoV-2) infection, also known as COVID-19 pandemic”, has developed an alarming unique circumstance worldwide.^1^ The pandemic has flipped the education equation for students, which may have affected their psychological and learning behaviors.^2^ In March 2020, several preventive control measures were implemented to control the spread of COVID-19 worldwide, including Saudi Arabia. These measures included an entire country lock-down for several months to combat the spread of the virus. Universities and schools were closed, students switched to virtual classes and were allowed to attend the campus for their practical lessons. This swift shift changes the regular sleep pattern and remaining activities of life.^2^

 Sleep medicine is an essential medical discipline^3^ and has been considered in the scientific field for many years. Poor sleep quality is one of the most common problems in modern times. The prevalence of sleep deprivation is increasing among people in both developing and modern societies.^4^,^5^ The quantity and quality of sleep play an essential part in individuals’ social, physical, and mental well-being.^6^ Sleep is a necessary and energizing body behavior that contributes to normal physiological and psychological functions, and it is difficult to change once compromised.^7^ The literature highlights the importance of sleep for overall health, memory, and higher cognitive consolidations.^8^

 Impaired sleep quality is seen in university students^9^ and affects the students’ academic performance,^10^ which is a significant concern for students, universities, and overall countries. This emphasizes the importance of assessing the sleep quality of university students, especially in the stressful situation during the COVID-19 pandemic. The present study aimed to evaluate and compare the sleep quality among medical and general science students during the COVID-19 pandemic.

## METHODS

### Study design and settings

*“*This questionnaire-based cross sectional study was conducted in the Department of Physiology, College of Medicine, King Saud University, Riyadh, Saudi Arabia,” during September-December 2020.

### Selection of Students and Data Collection

In this study, medical and science students at College of Medicine, College of Science, King Saud University, Riyadh, Saudi Arabia, were invited. The Science College includes the following divisions: Statistics, Mathematics, Zoology, Physics, Chemistry, Biochemistry, Botany, Microbiology, Astronomy, Geology, and Geophysics. The students’ emails and specialties were obtained from the Deanship of Admission and Registration Affairs office. The corroborated “self-administered electronic questionnaire” was circulated through email to the students after being selected randomly. We employed the power formula to calculate the sample size.

### Inclusion and Exclusion criteria

In this study, medical and science students at the “College of Medicine, College of Science, King Saud University, Riyadh” were invited. The participants with any previous known complaints of headache, anxiety, depression, sleep disturbances before the beginning of the COVID-19 pandemic were excluded from the study. The participants with a known history of chronic debilitating diseases, neurological, psychological disorders, and malignancy were excluded”.

### Questionnaire

The survey questionnaire consisted of 17 items focused on assessing sleep quality using the “Pittsburgh Sleep Quality Index (PSQI) scale.” The survey included an introductory page for informed consent, whether to contribute or not in the study. The students were given an option by clicking on the required option to their responses. No reward was presented to the contributors, and information was kept confidential. We used valid and reliable instruments to assess the sleep quality using the “Pittsburgh Sleep Quality Index (PSQI) Scale.”^11^

### Pittsburgh Sleep Quality Index (PSQI) Scale

PSQI is the most commonly rummage-sale instrument to assess sleep quality in the past month. It covers a wide range of gages relevant to sleep quality,^10^ including “subjective sleep quality, sleep latency, sleep duration, sleep efficiency, sleep disturbance, use of sleep medication, and daytime dysfunction.” For the PSQI scale, the elements of PSQI were scored out of three, with the minimum score being 0 and the maximum score being 3, where lower values indicated better sleep quality.^11^ While the cut-off point for the overall Global PSQI score was five. The PSQI has been validated in many languages with acceptable psychometric properties^12^ and is frequently used in clinical and research settings.^13^ The PSQI has also been validated in college students as cited in,^14^-^18^ including medical students.^19^,^20^ The psychometric properties of the PSQI suggest that it is a valid measure of “sleep quality, with a strong reliability coefficient (Cronbach’s alpha) of 0.83 for its seven components. The overall PSQI global score correlation coefficient for test-retest reliability was (r=0.87). The survey was distributed through emails. Initially, 1000 students were included, 782 of whom completed the study; the final included sample size was n=782.

### Ethical considerations

This study was approved by the “Institutional Review Board (IRB), Ethics Committee, College of Medicine Research Centre, King Saud University, Riyadh, Saudi Arabia,” approved the protocol (Ref #No.- E-20-5164).

### Statistical Analysis

The findings were analyzed by using (SPSS) software version 26.0 for Mac20. All categorical variables, including “age, gender, and occupation status,” were reported using frequency and proportions. The numbers and percentages were calculated. The response score was presented using mean and standard deviation. The various variables were compared using Chi-square, ANOVA, post-turkey hoc, independent samples t-tests, and Wilcoxon rank-sum tests as appropriate. A p-value < 0.05 was considered as significant.

## RESULTS

 The total number of medical and science students included was 782, out of whom 410 (52.4%) were medical students, and 372 (47.6%) were science students, including Physics, Chemistry, Mathematics, Statistics, Botany, and Zoology. Among medical students, 143 (34.9%) were in pre-clinical years (1st and 2nd), while 266 (64.9%) of them were in clinical years 3rd, 4th, and 5th-year medical students (Table-I).

**Table 1:** Sociodemographic characteristics of study subjects (n=782).

Characteristics	N (%)
***Age:***	
18-21	536 (68.5)
22-25	244 (31.2)
>25	2 (0.3)
***Gender***	
Male	271 (34.7)
Female	511 (65.3)
***Marital status***	
Single	777 (99.4)
Married	5 (0.6)
***Specialty***	
Medical students	410 (52.4)
Science students	372 (47.6)
***An academic year of medical students***	
Pre-clinical years (1st, 2nd)	143 (34.9)
Clinical years (3rd, 4th, 5th)	266 (64.9)
***Part-time job***	
Yes	17 (2.2)
No	765 (97.8)
***BMI***	
Underweight (<18.5)	105 (13.4)
Normal (18.5-24.9)	458 (58.6)
Overweight (25-29.9)	170 (21.7)
Obese (>30)	49 (6.3)
***Smoking status***	
Yes	61 (7.8)
No	721 (92.2)

### Sleep quality among science and medical students

Table-II demonstrated that out of 782 participants, 669 (85.55%) had poor sleep quality (≥5 Global PSQI score), while only 113 students had good sleep quality (<5 Global PSQI score). Out of 410 medical students, 336 (81.95%) had poor sleep quality, and 74 (18.05%) had good sleep quality. While out of 372 science students, 333 (89.5%) had poor sleep quality, and 39 (10.5%) had good sleep quality. Overall, it was seen that the sleep quality of science students was poorer (Mean Global PSQI score= 8.78) than their medical counterparts (mean Global PSQI score= 7.93) (Table-II).

**Table II:** Distribution of responses to different items of PSQI.

PSQI elements	Mean ± SD	Valid 0 N (%)	Valid 1 N (%)	Valid 2 N (%)	Valid 3 N (%)
Sleep Quality	1.431±0.088	105 (13.4)	338 (43.2)	236 (30.2)	103 (13.2)
Sleep Latency	2.09 ± 1.137	118 (15.1)	119 (15.2)	121 (15.5)	424 (54.2)
Sleep Duration	1.42 ± 0.990	197 (25.2)	153 (19.6)	341 (43.6)	91 (11.6)
Sleep Efficiency	0.055 ± 0.905	523 (66.9)	134 (17.1)	76 (9.7)	49 (6.3)
Disturbance	1.06 ± 0.499	72 (9.2)	590 (75.4)	118 (15.1)	2 (0.3)
Daytime Dysfunction	1.54 ± 0.871	95 (12.1)	271 (34.7)	312 (39.9)	104 (13.3)
***Global PSQI score***	8.34± 3.456	Minimum		Maximum	
		0		18	

### Sleep quality of medical students in relation to the academic year

Among the respondent medical students, 143 (34.9%) were in pre-clinical years (1st and 2nd), while 266 (64.9%) of them were in clinical years (3^rd^-5th). Table-IV shows that subjective sleep quality, sleep duration, and daytime dysfunction were poorer among pre-clinical students than clinical students (Table-III, IV).

**Table III:** Association between medical and general science and PSQI score.

PSQI elements	Medical students Mean ± SD	Science students Mean ± SD	t-test	Significance level.
Subjective Sleep Quality	1.40 ± 0.89	1.46 ± 0.87	-0.867	0.386
Sleep Latency	1.91 ± 1.2	2.29 ± 1	-4.737	0.000
Sleep Duration	1.31 ± 0.99	1.53 ± 0.98	-3.047	0.002
Habitual Sleep Efficiency	0.60 ± 0.92	0.51 ± 0.89	1.345	0.179
Disturbance	0.96 ± 0.47	1.18 ± 0.51	-6.350	0.000
Daytime Dysfunction	1.48 ± 0.86	1.62 ± 0.88	-2.212	0.027
Global PSQI	7.93	8.78	-3.440	0.001

**Table IV:** Association between the academic year of medical students and PSQI score

PSQI elements	Pre-clinical years Mean ± SD	Clinical years Mean ± SD	t-test	Significance level.
Subjective Sleep Quality	1.52 ± 0.94	1.34 ± 0.86	1.973	0.049
Sleep Latency	1.85 ± 1.24	1.94 ± 1.17	0.729	0.466
Sleep Duration	1.52 ± 1.03	1.21 ± 0.96	3.048	0.002
Habitual Sleep Efficiency	0.63 ± 0.90	0.58 ± 0.93	0.527	0.598
Disturbance	0.98 ± 0.48	0.95 ± 0.45	0.581	0.562
Daytime Dysfunction	1.59 ± 0.87	1.42 ± 0.85	1.992	0.047
Global PSQI	8.30	7.76	1.506	0.133

### Sleep quality in relation to demographic factors

Out of the selected cases with poor sleep quality (≥5 PSQI score), 32.1% were males, and 67.9% were females. The majority of the respondents, i.e., 58.6%, had normal BMI, 21.7% overweight, 13.4% underweight, and 6.3% obese. It was found that sleep disturbance and daytime dysfunction were poorer in students with higher BMI ranges.

## DISCUSSION

 The present study findings during this critical time of COVID-19 pandemic and online teaching situation spotlight the sleeping health of college students, especially medical and science students, concerning the increasing demands of online teaching. The present study results reveal that 85.5% of the students had poor sleep quality with a mean PSQI global score (8.356) among them, 50.2% were medical, and 49.8% were science students. Sleep is a physiological process and is essential to normal body functions. Medical and science students are the most vulnerable groups to sleep deprivation and poor sleep quality. Their depleting state has become a global concern since its consequences adversely affect academic life and personal peace.^21^ As per various studies, medical students suffer from significant sleep issues, resulting in lower sleep duration.

**Fig.1:**
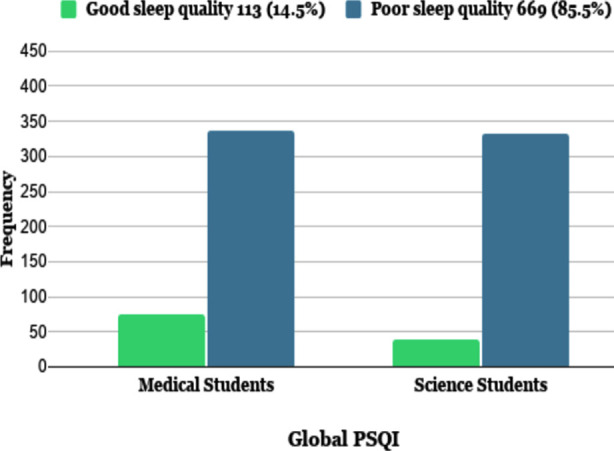
Global PSQI score for medical and science students.

 Multiple factors are affecting sleep deprivations.^22^ This study has incorporated tests based on the data collected through PSQI interpretation concerning various differentiations and factors.^22^ The results acquired concerning the quality of sleep based on the difference of gender reflect that females are prone to suffer from sleep deprivation more than males. It demonstrates the need for measures to promote sleep awareness programs with gender-specific motives and attempts. And these findings are consistent with the literature.^23^

 Another factor taken into consideration is the impact of BMI on sleep quality. The difference was found among obese or overweight respondents with issues like daytime dysfunction and disturbance. Even different studies also suggest that a higher weight can be associated with poor sleep quality.

 This study investigates the sleep quality among science students in the gulf region. There is a deficit of international literature concerning science students’ sleep quality. While among medical students, according to a study conducted at King Saud Bin Abdulaziz University, medical students suffer from poor sleep quality due to the extensive academic workload, clinical responsibilities, stressful work environment, and an extremely challenging lifestyle.^23^ We found that the COVID-19 online teaching situation had contributed to a pronounced increase in the percentage of poor sleep quality among medical students in Saudi Arabia. A study conducted among medical students in King Saud University published in 2012 reported that only 36.3% of the students had poor sleep quality^24^ in contrast with our findings in the same college during the COVID-19 pandemic, which reported that 81.95% of medical students suffered from poor sleep quality. These percentages are alarming and rarely presented in the literature; it may be due to the increasing demands of online teaching and perhaps the psychological stress during the COVID-19 pandemic. On an international scope, a study in Pakistan showed that 67.3% of medical students and 50.4% of non-medical students were classified as poor sleepers.^25^

 The assessment of sleep quality among medical students based on their academic year suggests that students in pre-clinical years have issues concerning subjective sleep quality, daytime dysfunction, and sleep duration. In other studies, the students in pre-clinical years face significant problems compared to others. As per a study conducted in a university in Brazil, the 1st and 2nd-year students suffered majorly due to adverse subjective sleep quality that is influenced due to the transition to medical curriculum and study schedule. It also highlighted the need for interventions among pre-clinical students to enhance their overall sleep quality.^20^

 To sum up, sleep disturbances among university students show an alarming new trend with unbearable consequences on the mental health of university students.^26^ Moreover, during COVID 19 pandemic, sleep disturbances were reported in students^26^ and healthcare workers.^27^ It is quite a critical issue and is essential to develop a positive environment while offering substantial support. Various measures such as counseling facilities, providing academic guidance, and promoting positive sleep patterns could benefit. Also, with collaborative efforts, this situation can be improved to pursue a healthy, productive future.^28^

### Study strengths and limitations

This sufficient sample size study compares sleep disturbance among university students during the COVID-19 pandemic. This study reported some interesting findings. However, this study has some limitations. First, the cross-sectional design limited the causality relationship interpretation, that COVID-19 is associated with sleep disturbance among university students. Second, our sample was restricted to King Saud University only. It would have been more appropriate to collect the data from other universities to extend our findings to the general population.

## CONCLUSION

 The COVID-19 pandemic has a negative impact on student’s mental health and sleep quality. The medical and science students showed alarming levels of sleep deprivation during the COVID-19 pandemic. The sleep deprivation among students may be due to the sudden change of pedagogy in education driven by the COVID-19 pandemic. The present study results may support implementing sleep education policies and academic counseling interventions to promote well-being in stressful conditions.

### Author’s Contribution:

**SAM, JMA, NFA:** Research conceptualization, Manuscript writing.

**WSA, AFA:** Data collection, analysis, and literature review.

All authors have read and approved the manuscript and are responsible and accountable for the accuracy or integrity of the work.
